# Professor Irving Fisher, President of the Committee of One Hundred on the “League of Medical Freedom,” and Other Important Matters—The Owen Bill—A Department of Health

**Published:** 1910-08

**Authors:** 


### CORRESPONDENCE

### Professor Irving Fisher, President of the Committee of One Hundred on the "League of Medical Freedom," and Other Important Matters —The Owen Bill—A Department of Health.

*Editor Buffalo Medical Journal:*

Sir: Buying newspaper space appears to be the latest method of opposing a popular movement. Within a few weeks, three enormous advertisements, by a so-called "League for Medical Freedom," have appeared in newspapers throughout the country urging people to write their congressmen in protest against the Owen bill for a National Department of Health. Much to the amusement of congressmen, these advertisements have resulted in numerous telegrams, based on the absurd idea that the Owen bill aims to regulate the practice of medicine.

The result may be to aid, rather than hinder, the progress of the health movement. Already newspaper editorials—such as that which recently appeared in the *New York Times*—resent the buncombe of these advertisements. But besides adding to the "gaiety of nations," this incident has created an appetite among the general public for more knowledge as to what this opposition signifies.

As president of the Committee of One Hundred on National Health, which was challenged in these advertisements, I wish to emphasise the following facts:

1. As bulletin 41 of the Committee of One Hundred indicates, the real strength of the opposition evidently comes from commercial interests, such as the quack medicine interests and others who have reason to fear the Pure Food and Drugs Act. While christian scientists and other "drugless" cults are denouncing drug doctors and denouncing a "medical trust" which does not exist, these cults themselves seem to be playing into the hands of a drug trust which does exist.

2. Under our Constitution, the Federal Government could not, if it would, regulate the practice of medicine.

3. The Owen bill contains no provision aiming to regulate the practice of medicine.

4. Section 8, in the Owen bill, the section authorizing the establishment of "chemical, biological, and other standards," has been eliminated entirely from the bill, although only a heated imagination could have construed this section as attempting to regulate the practice of medicine.

5. Senator Owen did not prepare his bill at the instance either of the American Medical Association or of the Committee of One Hundred on National Health. It is true, however, that the American Medical Association and the Committee of One Hundred heartily endorse the bill in preference to any of the other numerous health bills now before Congress.

6. The Committee of One Hundred on National Health is not a medical organisation. It did not originate with the American Medical Association, but with the American Association for the Advancement of Science. It is allied with the American Medical Association only in the same sense that it is allied with labor organisations, farmer organisations, life insurance companies and various other agencies which are working to improve public health.

7. While opposed to fraudulent quackery, which is always imposing "fake" medicines and cure-alls on the public, the Committee of One Hundred on National Health is not devoted to any particular school of medicine to the exclusion of others. Curiously enough, it was a christian scientist who moved the appointment of the Commititee of One Hundred. Many members of our committee have been noted for their independence of conventional medicine, among them being ex-president Eliot of Harvard University, Mr. William H. Allen, Mr. Bok, Editor of the *Ladies' Home Journal,* Mr. Horace Fletcher, Dr. Luther H. Gulick, President G. Stanley Hall, Mrs. John B. Henderson, Mr. S. S. McClure of *McClure's Magazine,* Dr. Richard C. Newton who has called attention to some merits in Osteopathy, and Mr. Nathan Straus. I may add that in my report on "National Vitality" to President Roosevelt, I put myself on record as favoring "medical freedom." I can endorse almost all of the position on that subject taken by Mr. Flower.

8. A department of health in America like the department of health in Germany or anywhere else will have better things to do than regulate the practice of medicine. It will regulate the misbranding of foods and drugs (there's the rub!), the pollution of streams, the inspection of meats and quarantine, and will obtain and distribute information in regard to health of human beings just as the Department of Agriculture does in regard to the health of hogs and cattle.

When the "League for Medical Freedom" suddenly appeared on the horizon, our movement had encountered substantially no opposition except among quacks and quack medicine proprietors. On the other hand, our movement has the support of the President, of both political parties, as expressed in their platforms, of scientific, philanthropic, medical and labor organisations and the Granges, as well as of the life insurance companies, of Dr. H. W. Wiley of the Bureau of Chemistry and of General Walter Wyman of the Public Health and Marine Hospital Service. The general public will find it hard to believe that these endorsements, especially the hearty endorsement of the life insurance companies, can be in the interests of a "medical trust."

. . . . . . . . . . . . . . .

IRVING FISHER,
President Committee of One Hundred on National Health.
460 PROSPECT STREET, NEW HAVEN, CONN.
June 11, 1910.

